# Learning transcriptional regulatory networks from high throughput gene expression data using continuous three-way mutual information

**DOI:** 10.1186/1471-2105-9-467

**Published:** 2008-11-03

**Authors:** Weijun Luo, Kurt D Hankenson, Peter J Woolf

**Affiliations:** 1 Department of Biomedical Engineering, University of Michigan, Ann Arbor, MI 48109, USA; 2 Bioinformatics Shared Resource, Cold Spring Harbor Laboratory, Cold Spring Harbor, NY 11724, USA; 3 Department of Animal Biology, University of Pennsylvania, Philadelphia, PA 19104, USA; 4 Bioinformatics Program, University of Michigan, Ann Arbor, MI 48109, USA; 5 Department of Chemical Engineering, University of Michigan, Ann Arbor, MI 48109, USA

## Abstract

**Background:**

Probability based statistical learning methods such as mutual information and Bayesian networks have emerged as a major category of tools for reverse engineering mechanistic relationships from quantitative biological data. In this work we introduce a new statistical learning strategy, MI3 that addresses three common issues in previous methods simultaneously: (1) handling of continuous variables, (2) detection of more complex three-way relationships and (3) better differentiation of causal versus confounding relationships. With these improvements, we provide a more realistic representation of the underlying biological system.

**Results:**

We test the MI3 algorithm using both synthetic and experimental data. In the synthetic data experiment, MI3 achieved an absolute sensitivity/precision of 0.77/0.83 and a relative sensitivity/precision both of 0.99. In addition, MI3 significantly outperformed the control methods, including Bayesian networks, classical two-way mutual information and a discrete version of MI3. We then used MI3 and control methods to infer a regulatory network centered at the MYC transcription factor from a published microarray dataset. Models selected by MI3 were numerically and biologically distinct from those selected by control methods. Unlike control methods, MI3 effectively differentiated true causal models from confounding models. MI3 recovered major MYC cofactors, and revealed major mechanisms involved in MYC dependent transcriptional regulation, which are strongly supported by literature. The MI3 network showed that limited sets of regulatory mechanisms are employed repeatedly to control the expression of large number of genes.

**Conclusion:**

Overall, our work demonstrates that MI3 outperforms the frequently used control methods, and provides a powerful method for inferring mechanistic relationships underlying biological and other complex systems. The MI3 method is implemented in R in the "mi3" package, available under the GNU GPL from  and from the R package archive CRAN.

## Background

A major challenge in systems biology is to infer mechanistic gene interactions from high throughput microarray data [1,2]. Underlying this challenge is the problem to find causal or regulatory relationships between genes. Robust solutions to this problem would provide us with a transcriptomic map of a genome that allows us to accurately predict the effect of gene perturbations.

Previous efforts to detect mechanistic relationships from gene expression data can be broadly divided into linear correlation and probability based methods. Linear correlation based methods, such as clustering [3,4], correlation networks [5,6] and graphical Gaussian models [7], have a long and fruitful history in statistical modeling and bioinformatics. These linear methods are computationally fast and relatively easy to interpret. However, a key limitation with these methods is that they assume linear relationships between variables. While some components of any transcriptional regulatory network are linear, nonlinear events such as OR, AND, and XOR type transcriptional regulation are relatively commonplace [8]. These nonlinear interactions would not be captured with a linear model, leading to spurious relationships between variables.

Probability based methods have also been used to detect relationships between genes. These probability methods include Probabilistic Boolean Networks (PBN) [9,10], Bayesian networks [11-14] and mutual information networks [15,16]. Probability based methods can capture both linear and nonlinear regulatory relationships and are noise tolerant. However, many of the current probability based tools used in systems biology suffer from the following three limitations: (1) data discretization [9-14,16], (2) pairwise testing [15,16], (3) emphasis on correlation over causality [11,12,14,17]. To transform continuous data into a more easily computable form, most probabilistic methods require the data to first be discretized into a finite number of bins, such as high, medium, and low [9-14,16]: The number of bins used in discretization is difficult to choose, and is generally selected at some consistent yet arbitrary point. Unfortunately, different binning procedures can produce different analysis results [12], suggesting that the act of binning alone introduces errors into the analysis. Methods that search for pairwise associations only focus on a single relationship between regulator and target at a time. Pairwise association networks have been created using classical mutual information [15,16]. However, simple pairwise relationships are likely less common than multivariate relationships in real biological systems, as the expression of most genes is regulated not by a single gene but more likely by multiple genes. Methods that allow multivariate interactions such as Bayesian networks or some fuzzy logic approaches [18] are inherently superior in this respect.

A final challenge in creating mechanistically predictive transcriptional models is the ability to identify not just correlative but also causal models. For the definition of causal relationship, we adopt the notion of probabilistic causation [19]. Informally, event A (probabilistically) causes event B if and only if A's occurrence alters (increases or decreases) the probability of B. This sometimes reflects imperfect knowledge (noise data) of a deterministic system but more frequently suggests a stochastic nature of the causal system under study. Although difficult, causal relationships have been learned properly from non-sequential observational data [20,21]. Probabilistic graphical modeling methods like Bayesian networks have been used to infer causal models from gene expression data [12,14]. (More details of the causality presentation using directed graphs [17] are given in Additional file 1: Supplementary Figure 2 and Supplementary Note 3.) However, many probabilistic approaches are able to make correlative networks but not necessarily causal networks [11,12,14,17]. Their multivariate scoring metrics such as conditional probability and mutual information are still generalized two-way correlation between the target and the parent set. Similar to the classical two-way metrics, these generalized correlations alone cannot differentiate between a causal versus confounding (merely correlative but non-causal) parent set. True causal relationships like genetic regulation feature positive higher order interaction [22,23], the non-additive effect above the sum of the lower order interactions [23]. For instance, for regulation involved two regulators such as OR, AND, XOR type relationships, two regulators together account for much more in the target than they individually can (Additional file 1: Supplementary Table 5). Intuitively such non-additive effect can be described as coordination or synergy between parents (with respect to the target, more description in Methods). On the other hand, confounding models commonly have no or negative higher order interaction (redundant parents, see the results). We propose that with such high order interaction considered, we can better differentiate true causal model versus confounding models.

In this work, we demonstrate a novel algorithm that attempts to overcome all three limitations using a continuous high order mutual information based scoring metric we call MI3 (Mutual Information 3, details in Methods part). Note that continuous two-way mutual information has been described previously [24]. High order interaction information (an extension of mutual information) has been employed to model complex interactions [22,23,25]. However, both two-way mutual information and high order interaction information are symmetric and as such unable to make causal statements. MI3 combines 3rd order interaction information with the asymmetric mutual information between target and regulator set to account for the direction of regulation. MI3 is novel as a combinatorial probabilistic metric and an integrated statistical learning method.

In this work, we compare MI3 to other probability based methods quantitatively and qualitatively using synthetic data where the true model is known. Next we apply MI3 and control methods to reconstruct regulatory networks centered at the transcription factor MYC from a published high throughput microarray dataset [15]. The learning results are then evaluated numerically and biologically.

Learning MYC centered transcriptional regulatory network represents an ideal test case for MI3 as MYC is a well characterized transcriptional regulator that acts in tandem with a finite set of co-effectors and regulates the expression of a large group of genes [26-28]. MYC has been well investigated [27,29,30] and online databases of MYC targets [31] are available for validation purpose. Despite these efforts, many cofactors and targets remain unidentified, and corresponding regulatory mechanisms unknown [15,26,27,29]. As a result, an integrated understanding of MYC dependent transcriptional regulation has remained out of reach [15,26,27,29,30]. In this study, we use MI3 to derive an accurate transcriptomic map surrounding MYC from the same gene expression dataset used to identify MYC targets [15]. The approaches used here are general and can be directly used for any transcriptional regulator given sufficient gene expression data.

## Results

### MI3 validation with synthetic data

**Figure 1:**
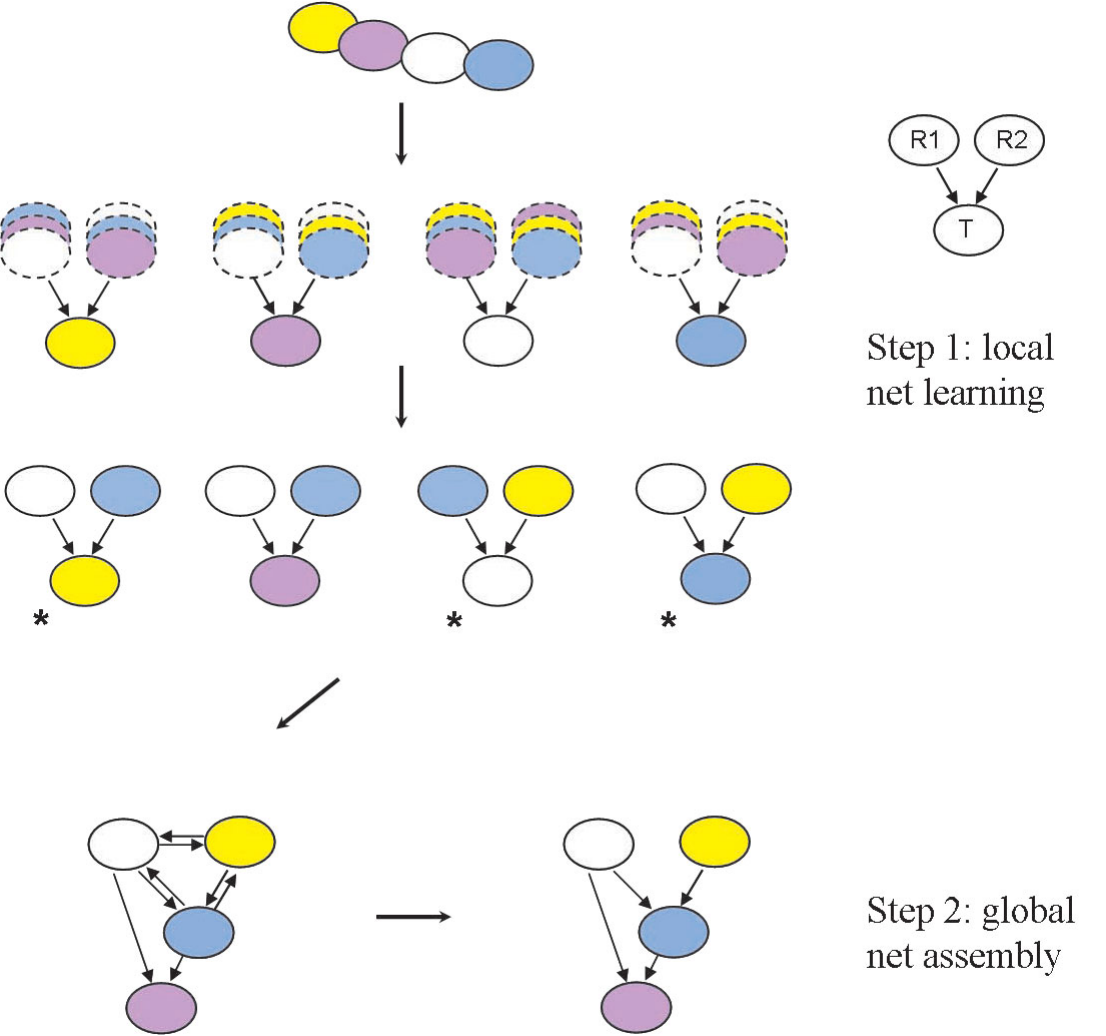
**A schematic view of the network inference procedure for MI3 and control methods.** We learn gene regulatory networks in two steps: (1) learn local regulatory network for each of the interesting nodes through an exhaustive search; (2) assemble local networks up into a unified network if needed. In the step (2), we may need to reconcile the conflicting local structures (labeled by *) if there are any, mainly the two way edges and cycles. More details of the procedure are described in Methods part. In this work, the key difference between different methods is the score metric being used rather than the network inference procedure. For a fair comparison between scoring metrics, we simple assemble the local networks up without the reconciliation of conflicts in step (2).

We validated MI3 against other commonly used methods listed in Table 1, including a discrete version of MI3 (dMI3), two-way mutual information (MI2) and a log conditional probability score used in Bayesian network (BN) learning. Each control method represents one of the three major issues described in the Introduction, with details given in Table 1. Learning was carried out using data sampled from a synthetic regulatory network, described in Additional file 1: Supplementary Figure 1 and Supplementary Table 1, where the true network structure is known. We learned the best two-parent regulatory model (Fig. 1) for each dependent node (u1–u6) by exhaustively searching through each possible parent set and scoring with each metric.

**Table 1:** MI3 and control methods evaluated and compared using the synthetic data.

**Method**	**Metric**	**Description**	**Performance Rank**	
			
			***Synth***	***Real***^#^
MI3	2*I(T;R1, R2)-I(T;R1)-I(T; R2) = I(T; R1|R2)+ I(T; R2|R1)	The sum of Correlative and Coordinative Criteria, which equals to the conditional mutual information between the target gene and the each regulator given the other regulator	1	1
dMI3	2*I(T;R1, R2)-I(T;R1)-I(T; R2)	Discrete version of MI3, control score to show the strength of continuous mutual information	3	2
Bayesian network (BN)	logP(T | R1, R2)^†^	Log conditional probability, control score which maximize correlation of the parent set to the target, while ignores the interaction between R1 and R2	2	3
Two-way MI (MI2)	I(T;R1)+I(T;R2)	Control two-way mutual information score to show the strength of three-way metric	4	4

Note that each control method compares to and validates MI3 in one of the three major aspects described in Introduction: data discretization (dMI3); pairwise testing (MI2); emphasis on correlation over causality (BN). All scores are calculated based on continuous nonparametric probability density estimation, except dMI3 based on discretization using 5 bins of equal size.

† In this paper, log conditional probability and BN are used interchangeably.

# Performance rank for real data experiment is based on qualitative comparison.

**Figure 2:**
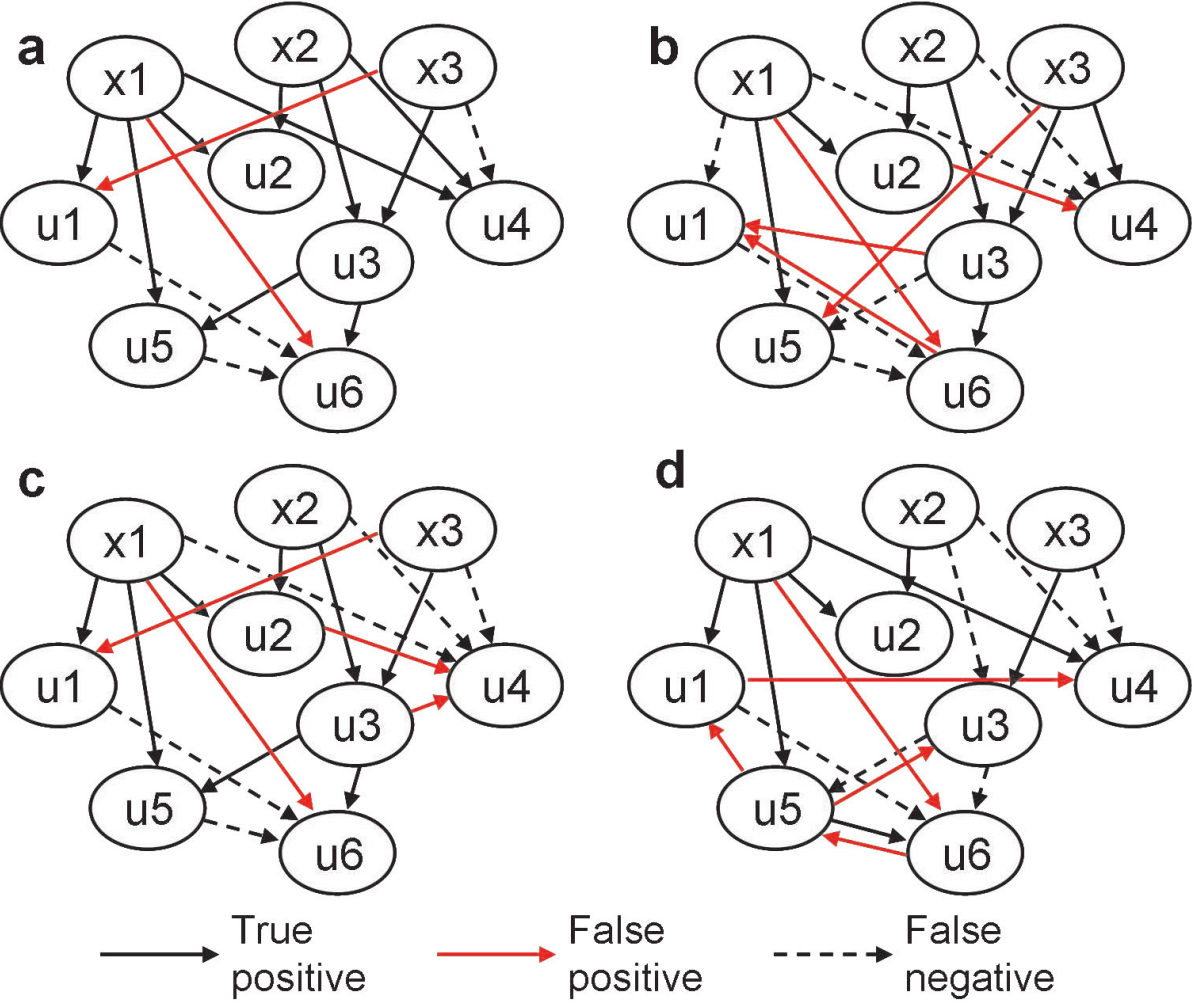
**Networks inferred by MI3 or control methods from a 350-sample synthetic dataset using the following 4 scoring metrics: (a) MI3, (b) dMI3, (c) BN (log conditional probability) and (d) MI2.** The best two parent model for each target gene was selected by using different methods and compared to true models. Here our interesting nodes are all the dependent nodes, u1–u6. Local regulatory networks are learned on these nodes and then assembled. When there is no information on dependent versus independent nodes, local networks are learned for all nodes including x1–x3. Conflicting local structures can be resolved in step (2) of Figure 1. For instance, the best two parents for x1 are u3 and u5, which conflicts with the local model for u5 whose parents are x1 and u3. Such conflicts were solved easily based on MI3 score, u3+u5->x1 scores 1.07 while x1+u3->u5 scores 1.49; hence the latter is the true model. The results remained essentially the same for MI3, BN and dMI3, but not for MI2.

The resulting best scoring network from a representative experiment is shown in Figure 2. Using the MI3 score, we recovered the true models for all dependent variables with exactly two parents, including u2, u3 and u5. For variables with fewer or more than two parents, i.e. u1, u4 and u6, MI3 detected the best two-parent representative of the true models. Continuous MI3 outperformed dMI3 as dMI3 identified poor models for u1, u4, and u5. The BN tended to select confounding yet correlative models with low or negative coordination (parents overlapping in their correlation with the target) between the two parents. For example, the BN score selected u2+u3 and x3+u2 over x1+x2 as the top 2 models for u4. Therefore, the coordinative component in MI3 is necessary to differentiate the true parent set from the confounding set. Compared to MI2, MI3 as well as log conditional probability consistently gave more accurate models whenever there was a difference, demonstrating their advantage in capturing higher order relationships. The existence of two way edges or edges with reversed direction showed that MI2 could not identify direction of causality between variables. In addition, the two parents for nodes u1, u4, u5 and u6 picked by MI2 have highly negative coordination with each other. These results demonstrate that, among the methods tested, MI3 most accurately identified the underlying regulatory network for both linear and nonlinear relationships between variables (Additional file 1: Supplementary Table 1).

**Figure 3:**
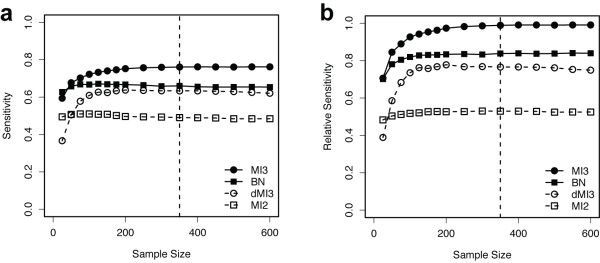
**Sensitivity curves for MI3 versus control methods in learning two-parent models from the synthetic dataset.** (a) Average absolute sensitivity of the 4 methods to recover the known network. (b) Average relative sensitivity of the 4 methods to recover the known network given that only two parents are possible for each dependent node. Vertical dashed lines marked sample size of 350 used in Figure 2, which is similar to the experimental sample size used for the MYC study.

Next we quantitatively compared the performance of MI3 to other commonly used methods in terms of both sensitivity (ratio of correctly inferred interactions among all true interactions) and precision (ratio of correct interactions among all inferred interactions) [15]. In Figure 3, only sensitivity curves are shown because the precision curves are essentially the same but shifted. Figure 3a provides the absolute performance, while 2b shows the relative performance. The relative performance is a more meaningful comparison, given that the number of parents was fixed, although both results are quite similar. The absolute sensitivity and precision MI3 algorithm achieved were 0.77 and 0.83 respectively (Figure 3a), and the relative levels are both 0.99 (Figure 3b). In this comparison, MI3 consistently outperformed dMI3 across all different sample sizes. Also MI3 was more robust than dMI3 in that the sensitivity and precision curves have smaller error bars (standard deviation not shown for better plot view). In addition MI3 always outperformed the correlative BN. MI2 consistently demonstrated the lowest performance by a large margin as long as the sample size was greater than 25. All methods reached a plateau at ~250 samples, indicating that the 350 (or 336 for real data) sample default used in this paper is appropriate for all 4 methods to learn two parent regulatory models (3 nodes). Finally, all four methods were ranked in terms of performance in Table 1. Overall, MI3 always gave the highest true positive and the lowest false positive rate, and significantly outperformed all control methods (p-value = 4.45 × 10^-11^), details of statistical tests shown given in Additional file 1: Supplementary Table 2.

While experiment with the above-mentioned small synthetic network clearly proved the principles of MI3 method, we also scaled up to a synthetic network that has the size of large gene regulatory networks yet still allows exhaustive search of two-parent models. This large synthetic network with 99 nodes and 165 edges was created by tiling 11 copies of the small network (Additional file 1: Supplementary Figure 1) plus 22 cross-tile edges [32]. Experiments with data sampled from this large synthetic network yielded similar results (data not shown) to those from the small synthetic study above.

### MI3 applied to high throughput microarray data

We used MI3 and control methods to infer regulatory network centered at MYC transcription factor from a human B cell microarray dataset. Note that the same dataset had been generated and used for identifying MYC target genes by another group [15]. Instead of doing an exhaustive search of co-regulator pairs for each target as in the synthetic data, we fixed one of the regulators to be MYC and the target to be a known MYC target, and searched for the second regulator. This constraint imposed by our specific biological focus made the analysis more tractable and our results more testable, because we only need to select and test the second regulator (more details given in Additional file 1: Supplementary Note 5). Notice that this simplified problem is a sub-case of the synthetic problem. We are still using the same scoring metrics (Table 1) and following the same procedure (Figure 1), except that one parent node is fixed by introducing extra literature data. In this sense, all methods are still comparable. Experiments with synthetic data showed that such simplification does not change the final results as long as we are introducing a real parent of the target with enough marginal dependency, i.e. I(T;R1) > 0.3, for MI3, dMI3 and BN. For MI2, fixing R1 = MYC does change the results, but it makes sense when taken as prior knowledge introduction. We pre-filtered MYC targets, T, with I(T; MYC) ≥ 0.3 to prevent bias upon fixing R1 = MYC, and to speed up analysis similar to candidate parent set selection in the sparse candidate algorithm [33].

The verified targets were retrieved from the MYC Target Gene Database [31] available online [34]. After pre-filtering using the constraint I(T; MYC) ≥ 0.3, 368 MYC targets remained as shown in Additional file 1: Supplementary Table 3. For each filtered target of MYC we selected top 5 cofactor (R2) models using MI3 or control methods. Because for each target gene, there are usually multiple models which score almost the same and are equally interesting biologically. For example, several coregulated MYC cofactors are involved in regulation of a target gene, any one of them can be selected as the best R2. Or multiple genes in a pathway/complex represent the same regulatory action equally well, all of them are sensible coregulators for a MYC target. This is slightly different from the synthetic experiment, where only there is 1 true or best model for each target. Nonetheless, keeping only top 1 model led to almost the same lists of most frequently selected cofactor (Additional file 1: Supplementary Table 4) as the list based on top 5 models (Table 2), except that the number of targets mapped to individual cofactors was smaller. All other comparisons between MI3 and control methods led to the same results when top 1 models were used (not shown).

**Table 2:** Top 10 most frequently selected coregulators for the 368 verified MYC targets using different methods.

Method	MI3		dMI3		BN		MI2	
***Rank\R2***	***Symbol***	***Targets***	***Symbol***	***Targets***	***Symbol***	***Targets***	***Symbol***	***Targets***
1	ARPC1B	45	**PSIP1**	46	**HAT1**	23	CTPS	29
2	TRIP12	45	FNBP1	42	**GTF2A2**	15	JTV1	24
3	**ASH2L**	41	MRPL28	28	PSMD14	14	MRPL3	23
4	**GCN5L2**	35	RAB33A	23	PSMA4	13	**SSRP1**	21
5	**SHOC2**	25	HSPB1	22	SFRS1	13	TPX2	20
6	**CSK**	23	TPP2	21	PSMA3	12	PSMB7	19
7	**ZNF143**	23	ANKMY2	18	ADRM1	11	RFC4	19
8	FNBP1	22	CD59	18	**DNMT1**	10	**MCM7**	18
9	**MIZF**	22	KIAA0922	17	CCT5	10	**HAT1**	18
10	**CBX1**	19	**SIAH2**	17	WDR62	10	HSPC111	17

Top 5 highest scoring cofactors are counted for each target. Cofactors in bold font are involved in MYC dependent or general transcriptional regulation, those in italics are in the list of 368 verified MYC targets with I(T; MYC) ≥ 0.3.

**Figure 4:**
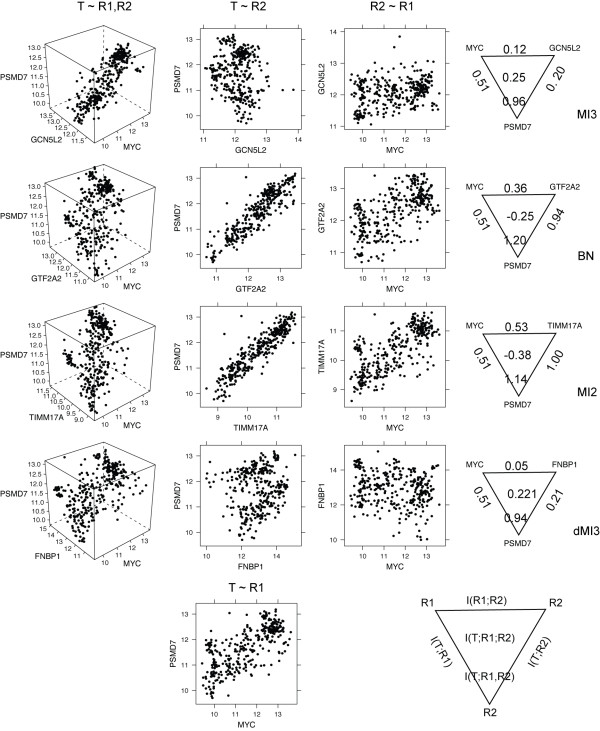
**Two-way and three-way gene expression patterns and mutual information for representative top two-parent models inferred by MI3 and control methods for T = PSMD7 given R1 = MYC.** The first three columns show the three-way and two-way gene expression patterns, and the fourth column the mutual information triangles. The bottom row shows the two-way expression pattern for PSMD7-MYC and the legend for mutual information triangle. This figure gives a concrete example for the difference between MI3 and control scores, echoing the results in Figure 5. For high throughput gene expression data, the BN and MI2 metrics both pick up models with high mutual information between parents and between either parent and the target. MI3 selected relationships with slightly lower I(T;R1, R2) but I(T;R1;R2) much higher than the BN and MI2 metrics.

MI3 and dMI3 selected models with significant coordination I(T;R1;R2), whereas the BN and MI2 selected models with high two-way dependency or I(T;R2) (note that I(T;R1) is constant because R1 is fixed to MYC) shown by Figure 4, 5. Models inferred by all methods showed distinct patterns when plotted in three dimensional space (T~R1, R2 in Figure 4). These patterns suggest that two parents together explain the target expression well. The difference is that BN and MI2 models showed distinct two dimensional patterns as well (T~R1 and R1~R2 in Figure 4), while the MI3 and dMI3 models did not. What MI3 and dMI3 captured are 3-way interactions in that neither of the two parents alone can describe the target well enough. In contrast, the relationships BN and MI2 captured are essentially two-way, and as such do not require both parents. This outcome is not surprising in that the MI3 metric favors strong three way interactions, while the BN and MI2 methods have no such favor and as such would be expected to include confounding two-way models more frequently.

**Figure 5:**
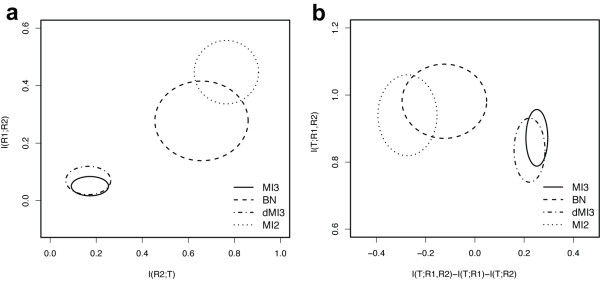
**Two-way and three-way mutual information distributions for top models selected by MI3 and control methods. **For each MYC target gene, the top 5 R2 or MYC cofactors were selected by applying different scoring metrics to the microarray dataset generated by Basso et al [15]. (a). I(R1;R2) vs. I(R2;T), i.e. two way mutual information between R2 and R1 or T, (b). I(T;R1, R2) vs. I(T;R1, R2)-I(T;R1)-I(T;R2), i.e. the correlative and coordinative components of MI3 score for the top 5 models selected by MI3 or control methods. Each ellipse represents the distribution of top 5 models in the specified mutual information coordinates, with mean as center and standard deviations as width and height. Note that I(R1;T) scores are the same for all methods hence not shown in (a).

**Figure 6:**
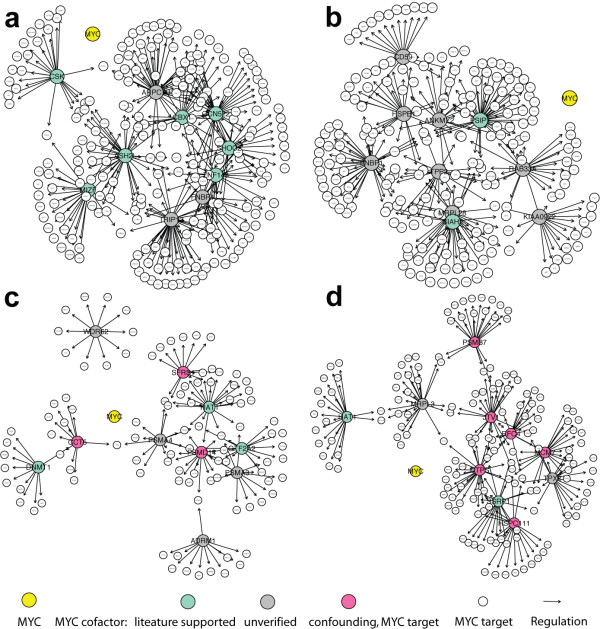
**The transcriptional regulatory networks centered at MYC transcription factor.** Networks included the top 10 most frequently selected MYC cofactors by using MI3 or control methods and the corresponding target genes (transparent). (a-d) are networks inferred by MI3, dMI3, BN and MI2 respectively. Regulators are large nodes and targets are small transparent nodes. Node colors indicate the identity where yellow is MYC, aquamarine are the cofactors involved in MYC dependent or general transcriptional regulation according to literature, gray are unverified cofactors, pink are confounding cofactors that are actually verified MYC targets. Edges represent transcription regulation. Note that all edges from MYC to targets are hidden for clarity.

BN and MI2 models had low or negative 3-way coordination, and are likely confounding models. The relationship R2~R1 is similar to T~R1 and T~R2 follows a nearly perfect linear pattern (Figure 4). Such high similarity between R2 and T is unlikely true regulation but rather coregulation (by other genes) relationship when considering various other factors affecting the target gene expression that are not counted by transcription level of the regulator(s), such as mRNA to protein translation, protein modification, and localization of the regulator. In other words, real regulators do not correlate so well with their targets (like MYC and its targets, T~R1 in Figure 4). We expect that the R2 factors predicted by the BN and MI2 methods is most often another MYC target tightly coregulated with T instead of a coregulator, and indeed many top R2 are MYC targets (Table 2 and Figure 6, more description next).

Next we collected the top 5 cofactors and ranked each cofactor according to its frequency of being selected. Table 2 lists the top 10 most frequently selected cofactors using the four methods. Transcriptional regulatory networks centered at MYC were constructed based on the top 10 cofactors and corresponding targets, as shown in Figure 6. Literature validation was focused on these top 10 cofactor lists (Table 2).

Top 10 cofactor based transcriptional regulatory networks constructed by MI3 and dMI3 were larger and covered more MYC targets than the networks created by BN and MI2 (Figure 6). Out of 368 MYC targets, MI3 places 56.3% of these targets while dMI3 places 51.6%, BN places 26.9%, and MI2 places 41.8% of the targets. In other words, more MYC target genes are regulated by the top 10 mechanisms inferred by MI3 or dMI3, which is more consistent with the current mechanistic understanding of MYC dependent transcription that MYC regulates a large number of targets (> 1000 verified) [26,31] as a global transcriptional regulator yet only interacts with a small set of cofactors (13 listed) [27,29].

Biologically, top 10 MYC cofactor list selected by MI3 was more consistent with the literature than the lists created by the control methods (Table 2). Seven out of ten MI3 top MYC cofactors are involved in MYC dependent or general transcriptional regulation. GCN5L2 (known as human GCN5), ASH2L, MIZF, CBX1 (HP1 beta homolog Drosophila) are chromatin structure modifiers, which change chromatin structure around target genes through chemical modification hence activate or repress their transcription. Chromatin structure modification by GCN5L2 and similar enzymes is a well documented mechanism for MYC dependent transcriptional regulation [27,28,35,36]. ZNF143 [37] and MIZF [38] are transcriptional factors. CSK phosphorylates and activates GSK-3beta directly [39] and indirectly [40], while GSK-3beta phosphorylates, deactivates MYC and promotes its degradation [27]. SHOC2 complexes with Ras and Raf and enhances MAP kinase activation [41,42], which in turn positively regulates MYC stability/activity by phosphorylation [27]. In contrast, only 2 (PSIP1, SIAH2), 3 (HAT1, GTF2A2, DNMT1) and 3 (SSRP1, MCM7, HAT1) top 10 MYC cofactors selected by dMI3, BN and MI2 respectively are transcriptional regulators based on Gene Ontology and literature.

Moreover, 3 (SFRS, CCT5, PSMD14) and 6 (CTPS, JTV, PSMB7, RFC4, MCM7, HSPC111) top 10 MYC cofactors selected by BN and MI2 respectively are actually from the 368 verified MYC targets. Other top 10 cofactors selected by BN and MI2 are likely 'unverified' MYC targets given that they either share function annotations or have similar expression profile with these questionable cofactors. In other words, BN and MI2 frequently produced confounding models where target genes were mistaken as MYC cofactors, while MI3 and dMI3 produced no confounding models. In Figure 6d, the two-way edges between red nodes suggest that MI2 not only confounded coregulators with targets, but also failed to tell the causal direction of the relationships. Combined with numerical comparison in Figure 4, 5, these biological results show that unlike BN and MI2 scores, MI3 score effectively differentiates true causal models from confounding models because it takes the interaction between regulators into account.

## Discussion

In this study, we have used MI3 to identify mechanistically plausible relationships from gene expression data. For synthetic data, MI3 recovered all true two-parent models, or the best representatives of the true models, and showed superior performance over the commonly used probability based methods including Bayesian networks and classical two-way mutual information. For experimental data, MYC cofactors identified by MI3 are either true or strongly supported by literature, while cofactors identified by control methods make little sense. Notably, the same microarray dataset has been used to identify MYC targets based on two-way mutual information [15].

MI3 uses three strategies to improve its predictions. First, MI3 does not require data discretization, and as such retains more of the information in the data. This continuous method enhanced the learning quality significantly, as shown by the synthetic example in Figure 2, 3. Second, we extended classical two-way mutual information to three-way, which allows MI3 to capture more complex relationships between regulators and targets. Third, the MI3 score considers high order interaction or coordination and better differentiates causal relationships from confounding relationships as was shown by both the synthetic and MYC problems (Figure 2 and 6).

MYC cofactors predicted by MI3 details agree with the established literature. Notably, four of the top 10 cofactors selected by MI3 are chromatin structure modifier genes, suggesting that chromatin structure modification is the primary mechanism for MYC dependent transcriptional regulation. This inference is directly supported by the independent experimental results of Knoepfler et al (21), which provides further evidence of the role of MYC on chromatin structure modification via histone acetylation and methylation. Among the top MYC cofactors identified by MI3, GCN5L2 [27,28,30,43], CSK [27,39,40], and SHOC2 [41,42] are known or presumed coregulators for MYC transcriptional activity. All other seven MYC cofactors selected by MI3 are novel, although their connections to MYC or transcription are well documented. All these results demonstrate that MI3 is an accurate and powerful method to infer regulatory models from microarray data. In contrast, top MYC cofactors inferred using control methods make much less sense biologically. Fewer of them are known transcriptional regulators and none of them is directly connected to MYC function. The fact that multiple MYC targets were mistaken as top MYC coregulators suggests that BN and MI2 methods have difficulty inferring true causal relationships from high throughput gene expression data. Generally speaking, it is sensible that some MYC targets can be its cofactors as seen in feedback loops. However, it is not likely that these MYC targets taken as co-regulators are real co-regulators because of feedback loops, since almost all of them are not functionally related to transcriptional regulation or MYC regulation activity. Similar confounding regulators were selected by control methods in the synthetic example (Figure 2). Figure 4, 5 show why such confounding models occurred. There are likely feedback loops in MYC regulation, however these feedback relationships could only be identified with knockout data or time series data. In this work we only consider the general case where non-sequential observational gene expression data are available.

Learning from high throughput microarray data was different from learning from the small synthetic dataset. Differences between methods were larger for the microarray data (Figure 6 and Table 2), compared to the synthetic experiment (Figure 2). For the microarray data, MI3 and dMI3 were closer, whereas for the synthetic data BN and MI3 were closer (Table 1). This change in ranking suggests that the coordinative component was more significant than the difference made by using continuous versus discrete metric (MI3 vs. dMI3) or 3-way versus 2-way metric (BN vs. MI2) for microarray data, but not for synthetic data. These differences between microarray data and synthetic data can be ascribed to the fact that large numbers of highly correlative confounding models exist for the microarray data due to the large number of variables (genes), especially coexpressed genes, while the synthetic data contained relatively fewer possible confounding models.

The high order mutual information framework presented here is generally applicable, although we have only described and used three-way mutual information. The same set of strategies can be used to model arbitrarily high order relationships. To learn a regulatory model with d dimensions or nodes (1 child with d-1 parents) by exhaustive searching through a system with v variables, we need ~10*5^d ^data samples for nonparametric probability density estimation [44-46], and computation time is O(v^d^). Although 10*5^d ^is conservative compared to sufficient sample size indicated in the performance curve, ~250 for d = 3 (Figure 3), undoubtedly, both the required dataset size and computational time exponentially increase with d. Therefore, 4-way or 5-way relationships require more samples than currently available microarray chips.

Through the use of MI3 we have demonstrated that tailored probability based metrics can outperform more standard methods used in systems biology for identifying mechanistic regulatory relationships. We expect that future enhancements to these scoring metrics are possible to identify larger sets of regulators while making fewer assumptions during the analysis.

## Conclusion

MI3 is a novel method for learning probabilistic graphical models and addresses three common issues in previous methods simultaneously: (1) handling of continuous variables, (2) detection of more complex three-way relationships and (3) better differentiation of causal versus confounding relationships. Simulation experiments show that MI3 consistently and significantly outperforms the frequently used control methods such as discrete probabilistic networks, two-way mutual information networks and Bayesian networks. When applied to a human B cell microarray dataset, MI3 recovered cofactors for MYC transcription factor and revealed the major regulatory mechanisms involved in MYC dependent transcriptional regulation, which are directly verified or strongly supported by literature. Overall, MI3 provides a powerful method for inferring mechanistic relationships underlying biological and other complex systems.

## Methods

### MI3 algorithm

The MI3 algorithm is a novel three-way mutual information engine for local causal model inference. Our hypothesis is that gene expression regulation commonly involves more than 2 genes (i.e. more than 1 regulator gene) with higher order interaction, which can be faithfully captured by continuous higher order mutual information. The algorithm is limited to three-way mutual information (two regulators and one target) (Fig. 5), but the same method can be easily extended to higher order mutual information to model more complicated regulation mechanisms. Note that we call all types of mutual information involving 3 variables 3-way mutual information (Additional file 1: Supplementary Note 1), while three-way interaction information refers to I(T;R1;R2) only.

The MI3 scoring function has two parts, including correlative and coordinative information components. The correlative component measures the correlation between the target and the parent set, similar to other correlative probabilistic metrics such as log conditional probability for Bayesian networks.

Correlative component: I(T; R1, R2)

Here I is the mutual information function, T is the target gene, and R1 and R2 are the regulators as illustrated in Figure 1. Mutual information definition and high order extensions are describe in detail in the Additional file 1: Supplementary Note 1. Pairs of regulators accurately describing the expression of the target gene will score well by the correlative component.

The coordinative component measures the coordination effect between the regulators with respect to the target. Note this component is actually the third order interaction information between T, R1 and R2, i.e. I(T; R1; R2) [23], and is three-way symmetric.

Coordinative component: I(T; R1, R2)-I(T; R1)-I(T; R2)

The coordinative component of the score identifies how well pairs of regulators versus individual regulators predict the target (examples in Additional file 1: Supplementary Table 5). Confounding models commonly have a negative coordinative score because parents overlap in their correlation with the target. The coordinative component can be rearranged to I(T; R1|R2)-I(T; R1), suggesting that this component measures how much better R1 predicts T given R2 versus not given R2. The coordinative component provides a quantitative measurement for the well-known 'selection bias' (also called Berkson's paradox) [47] in statistics or the 'explaining-away phenomenon' in Bayesian network theory [48].

The MI3 score is the sum of the correlative and coordinative component.

MI3 score: 2*I(T; R1, R2) – I(T; R1)-I(T; R2) = I(T; R1|R2)+ I(T; R2|R1)

The symmetric coordinative component captures higher order interactions and differentiates causal relationships from confounding ones without telling the causal direction. The asymmetric correlative component determines the direction of the causal relationship. By merging these two components, the MI3 score considers connections between the regulators as well as dependency between child and regulators. The MI3 score can be rearranged and simplified to I(T;R1|R2)+ I(T;R2|R1). This rearrangement can be interpreted as the conditional mutual information between the target gene and the each regulator given the other regulator, which better shows the three-way nature of this score. The MI3 score is structurally different from yet related to other probability scoring metrics such as log based conditional probability used in Bayesian network learning logP(T|R1, R2) [11,12] and two-way mutual information I(T;R1)+I(T;R2) [15,16] (described in Table 1 and Additional file 1: Supplementary Note 2).

#### Network inference procedure

Regulatory network inference procedure based on MI3 is shown in Figure 1. We learn gene regulatory networks in two steps: (1) learn local regulatory network for each of the interesting nodes through an exhaustive search. When there is no list of interesting nodes, all nodes becomes interesting. (2) assemble local networks up into a unified network if needed. Similar to Bayesian networks, the gene regulatory networks learned by using MI3 is directed acyclic. In the step (2), we may need to reconcile the conflicting local structures (labeled by *) if there are any, mainly the two way edges and cycles. We solve conflicting local structures based on their scores. For instance, in Figure 1, the local models for the yellow, white and blue genes conflict. The local model for blue gene scores the highest based on MI3 (or control score), hence it is kept in the final network and two other conflicting models are discarded.

Note that the key difference between MI3 and control methods is the scoring metrics, less in the network construction procedure. For a fair comparison between methods, we keep the procedure for all methods the same as in Figure 1. For more details on how the local network was selected see Additional file 1: Supplementary Note 4.

MI3 is implemented in the statistical computing language R, and codes are available online [49].

### Nonparametric probability density estimation for continuous variables

To avoid discretizing our data to calculate mutual information, we have adopted a continuous method for mutual information calculation based on a classical nonparametric Gaussian kernel method in probability density estimation [44,45]. To estimate the probability density at a specific location, we used all our data points. First we calculate the probability density at an interesting location based on a Gaussian distribution centered at each data point (kernel), and then take the average of all these densities using the following expression:

$$f(x)=\frac{1}{N}\sum_{i=1}^{N}\frac{1}{{\left(2\pi \sigma^{2}\right)}^{d/2}}e^{\left(-\frac{{\left|x-xi\right|}^{2}}{2\sigma^{2}}\right)}$$

Here x is the position where probability density is to be estimated, and xi (i = 1,2,, N) is the ith data point, both x and xi are d-dimension vectors, σ is the standard deviation of the kernel Gaussian distribution. We used optimal bandwidth described by Scott [45]. Our experiments with uniform kernel (data not shown) showed the choice of kernel distribution makes little difference in probability estimation, as has been noted elsewhere [44]. The reason we chose to use a Gaussian kernel is that it is intuitive and the result probability density distribution is continuous and infinitely differentiable [44]. Data may be transformed into a uniform distribution [24] before the kernel density estimation to eliminate the potential effect of specific distributions. We found uniform transformation does help but the improvement is limited when the gene expression data are log transformed. This nonparametric kernel method as a mature strategy for probability density estimation, its performance has been established in the original works [44,45]. Dependence of the estimation error on bandwidth (σ), dimensionality (d) and sample size (N) of the problem has been detailed discussed too [44-46].

Following our description above, to calculate entropy and mutual information for continuous variables, we calculated a probability density estimate at the positions of sample data points, then took the sample mean of log probability density [24], to approximate the full integration. The probability density estimation was the most computationally intensive step for this work.

Nonparametric probability density estimation for continuous variables effectively eliminates the inaccuracies introduced by discretizing data. However, this method is computationally demanding, and requires a large sample size (N) [44-46]. Due to these limitations, we limited our MI calculation to 3 variables. Notice that the sufficient sample only depends on the number of relevant dimensions of the local models (3 nodes, Figure 1), and has nothing to do with the size of the total number of variables.

To compare our continuous approach to more commonly used discretization approaches, we used 5 bins of equal size.

### Generation of synthetic testing data

Synthetic data was used to validate our MI3 method as an example of a completely known gene regulatory network. We created a synthetic network structure with algebraic relationships between variables found in Supplemental Figure 1 and Supplemental Table 1 online. We sampled 25 to 1000 samples from this network to generate a set. At each sample size, the sampling-learning procedure was repeated 500 times to determine the average sensitivity and precision of MI3 and control methods. This model structure is designed to mimic a miniature gene regulatory system, with regard to the network size, overall and local structure, and dependency relationships.

### Gene expression data processing and annotation

A gene expression dataset of human B cells with 336 samples was used for our study. These data were collected on the Affymetrix HG-U95Av2 platform and published by another group [15]. The raw data in .CEL format was collected from Gene Expression Omnibus (GEO) and processed by using RMA [50] method implemented in Bioconductor [51] Affy package [52]. A up-to-date probe set definition (.CDF file) based on Entrez Gene sequence, Hs95Av2_Hs_ENTREZG_7, created by the Microarray Lab at University of Michigan [53,54], is used in place of the Affymetrix original probe set definition provided by Bioconductor [55]. The corresponding annotation data was generated with AnnBuilder package based on the latest release of public databases, including Entrez Gene, UniGene, PubMed of NCBI, Gene Ontology (GO) and KEGG.

For downstream analysis, all genes are included without discriminative filtering process based on magnitude of changes. The expression level for each gene is standard normalized before use.

### Statistical analysis for difference in the method performance

One-way ANOVA followed by a Tukey test was used to evaluate the performance of MI3 score in comparison to control scores in learning 2-parent models from synthetic data, as shown in Additional file 1: Supplementary Table 2. One-way ANOVA tested whether the performance of all four methods are the same and Tukey test exactly where (between the methods) the difference lies. Histograms (not shown) indicate that sensitivity or precision for each score closely approximates normal distribution, and this is especially true when sample size is large (number of experiments = 500). Hence, one-way ANOVA and Tukey test are appropriate statistical tests. Tukey tests were conducted for all potential pair-wise comparisons.

## Authors' contributions

WL and PJW conceived and designed the study; WL conducted the research and wrote the computer program; WL, KDH and PJW drafted the manuscript. All authors read and approved the final manuscript.

## Supplementary Material

Additional file 1 **Supplementary figures, tables and notes.** Click here for file

## References

[B1] Lockhart DJ, Dong H, Byrne MC, Follettie MT, Gallo MV, Chee MS, Mittmann M, Wang C, Kobayashi M, Horton H (1996). Expression monitoring by hybridization to high-density oligonucleotide arrays. Nat Biotechnol.

[B2] Schena M, Shalon D, Davis RW, Brown PO (1995). Quantitative monitoring of gene expression patterns with a complementary DNA microarray. Science.

[B3] Eisen MB, Spellman PT, Brown PO, Botstein D (1998). Cluster analysis and display of genome-wide expression patterns. Proc Natl Acad Sci USA.

[B4] Spellman PT, Sherlock G, Zhang MQ, Iyer VR, Anders K, Eisen MB, Brown PO, Botstein D, Futcher B (1998). Comprehensive identification of cell cycle-regulated genes of the yeast Saccharomyces cerevisiae by microarray hybridization. Mol Biol Cell.

[B5] Butte AJ, Tamayo P, Slonim D, Golub TR, Kohane IS (2000). Discovering functional relationships between RNA expression and chemotherapeutic susceptibility using relevance networks. Proc Natl Acad Sci USA.

[B6] Moriyama M, Hoshida Y, Otsuka M, Nishimura S, Kato N, Goto T, Taniguchi H, Shiratori Y, Seki N, Omata M (2003). Relevance network between chemosensitivity and transcriptome in human hepatoma cells. Mol Cancer Ther.

[B7] Schafer J, Strimmer K (2005). An empirical Bayes approach to inferring large-scale gene association networks. Bioinformatics.

[B8] Alon U (2007). An introduction to systems biology: design principles of biological circuits.

[B9] Shmulevich I, Dougherty ER, Kim S, Zhang W (2002). Probabilistic Boolean Networks: a rule-based uncertainty model for gene regulatory networks. Bioinformatics.

[B10] Shmulevich I, Zhang W (2002). Binary analysis and optimization-based normalization of gene expression data. Bioinformatics.

[B11] Hartemink AJ, Gifford DK, Jaakkola TS, Young RA (2001). Using graphical models and genomic expression data to statistically validate models of genetic regulatory networks. Pac Symp Biocomput.

[B12] Friedman N, Linial M, Nachman I, Pe'er D (2000). Using Bayesian networks to analyze expression data. J Comput Biol.

[B13] Sachs K, Perez O, Pe'er D, Lauffenburger DA, Nolan GP (2005). Causal protein-signaling networks derived from multiparameter single-cell data. Science.

[B14] Friedman N (2004). Inferring cellular networks using probabilistic graphical models. Science.

[B15] Basso K, Margolin AA, Stolovitzky G, Klein U, Dalla-Favera R, Califano A (2005). Reverse engineering of regulatory networks in human B cells. Nat Genet.

[B16] Butte AJ, Kohane IS (2000). Mutual information relevance networks: functional genomic clustering using pairwise entropy measurements. Pac Symp Biocomput.

[B17] Pearl J (2000). Causality: models, reasoning, and inference.

[B18] Woolf PJ, Wang Y (2000). A fuzzy logic approach to analyzing gene expression data. Physiol Genomics.

[B19] Hitchcock C (2002). Probabilistic Causation. http://plato.stanford.edu/entries/causation-probabilistic/.

[B20] Dupont WD (1978). Making causal inferences from observational data. Biometrics.

[B21] Winship C, Morgan SL (1999). The Estimation of Causal Effects from Observational Data. Annual Review of Sociology.

[B22] Mcgill WJ (1954). Multivariate Information Transmission. Psychometrika.

[B23] Jakulin A, Bratko I (2004). Quantifying and Visualizing Attribute Interactions: An Approach Based on Entropy. arXiv:csAI/0308002.

[B24] Steuer R, Kurths J, Daub CO, Weise J, Selbig J (2002). The mutual information: detecting and evaluating dependencies between variables. Bioinformatics.

[B25] Nemenman I (2004). Information theory, multivariate dependence, and genetic network inference. arXiv:q-bio/0406015.

[B26] Li Z, Van Calcar S, Qu C, Cavenee WK, Zhang MQ, Ren B (2003). A global transcriptional regulatory role for c-Myc in Burkitt's lymphoma cells. Proc Natl Acad Sci USA.

[B27] Adhikary S, Eilers M (2005). Transcriptional regulation and transformation by Myc proteins. Nat Rev Mol Cell Biol.

[B28] Knoepfler PS, Zhang XY, Cheng PF, Gafken PR, McMahon SB, Eisenman RN (2006). Myc influences global chromatin structure. Embo Journal.

[B29] Eisenman RN (2001). Deconstructing myc. Genes Dev.

[B30] Cowling VH, Cole MD (2006). Mechanism of transcriptional activation by the Myc oncoproteins. Semin Cancer Biol.

[B31] Zeller KI, Jegga AG, Aronow BJ, O'Donnell KA, Dang CV (2003). An integrated database of genes responsive to the Myc oncogenic transcription factor: identification of direct genomic targets. Genome Biol.

[B32] Tsamardinos I, Statnikov A, Brown LE, Aliferis CF (2006). Generating Realistic Large Bayesian Networks by Tiling. Proceedings of the Nineteenth International Florida Artificial Intelligence Research Society (FLAIRS Conference).

[B33] Friedman N, Nachman I, Pe'er D (1999). Learning Bayesian Network Structure from Massive Datasets: The "Sparse Candidate" Algorithm. Proceedings of the 15th Annual Conference on Uncertainty in Artificial Intelligence (UAI-99); San Francisco, CA.

[B34] The MYC Target Gene Database. http://www.myccancergene.org/site/mycTargetDB.asp.

[B35] Pal S, Yun R, Datta A, Lacomis L, Erdjument-Bromage H, Kumar J, Tempst P, Sif S (2003). mSin3A/histone deacetylase 2- and PRMT5-containing Brg1 complex is involved in transcriptional repression of the Myc target gene cad. Mol Cell Biol.

[B36] Ogawa H, Ishiguro K, Gaubatz S, Livingston DM, Nakatani Y (2002). A complex with chromatin modifiers that occupies E2F- and Myc-responsive genes in G0 cells. Science.

[B37] Schuster C, Krol A, Carbon P (1998). Two distinct domains in Staf to selectively activate small nuclear RNA-type and mRNA promoters. Mol Cell Biol.

[B38] Mitra P, Xie RL, Medina R, Hovhannisyan H, Zaidi SK, Wei Y, Harper JW, Stein JL, van Wijnen AJ, Stein GS (2003). Identification of HiNF-P, a key activator of cell cycle-controlled histone H4 genes at the onset of S phase. Mol Cell Biol.

[B39] Fan G, Ballou LM, Lin RZ (2003). Phospholipase C-independent activation of glycogen synthase kinase-3beta and C-terminal Src kinase by Galphaq. J Biol Chem.

[B40] Dominguez-Caceres MA, Garcia-Martinez JM, Calcabrini A, Gonzalez L, Porque PG, Leon J, Martin-Perez J (2004). Prolactin induces c-Myc expression and cell survival through activation of Src/Akt pathway in lymphoid cells. Oncogene.

[B41] Rodriguez-Viciana P, Oses-Prieto J, Burlingame A, Fried M, McCormick F (2006). A phosphatase holoenzyme comprised of Shoc2/Sur8 and the catalytic subunit of PP1 functions as an M-Ras effector to modulate Raf activity. Mol Cell.

[B42] Li W, Han M, Guan KL (2000). The leucine-rich repeat protein SUR-8 enhances MAP kinase activation and forms a complex with Ras and Raf. Genes Dev.

[B43] Liu X, Tesfai J, Evrard YA, Dent SY, Martinez E (2003). c-Myc transformation domain recruits the human STAGA complex and requires TRRAP and GCN5 acetylase activity for transcription activation. J Biol Chem.

[B44] Silverman BW (1986). Density estimation for statistics and data analysis.

[B45] Scott DW (1992). Multivariate density estimation: theory, practice, and visualization.

[B46] Scott DW, Wand MP (1991). Feasibility of Multivariate Density Estimates. Biometrika.

[B47] Grimes DA, Schulz KF (2002). Bias and causal associations in observational research. Lancet.

[B48] Pearl J (1988). Probabilistic reasoning in intelligent systems: networks of plausible inference.

[B49] The MI3 Algorithm R packages. http://sysbio.engin.umich.edu/~luow/downloads.php.

[B50] Irizarry RA, Hobbs B, Collin F, Beazer-Barclay YD, Antonellis KJ, Scherf U, Speed TP (2003). Exploration, normalization, and summaries of high density oligonucleotide array probe level data. Biostatistics.

[B51] Gentleman RC, Carey VJ, Bates DM, Bolstad B, Dettling M, Dudoit S, Ellis B, Gautier L, Ge Y, Gentry J (2004). Bioconductor: open software development for computational biology and bioinformatics. Genome Biol.

[B52] Gautier L, Cope L, Bolstad BM, Irizarry RA (2004). affy – analysis of Affymetrix GeneChip data at the probe level. Bioinformatics.

[B53] Dai M, Wang P, Boyd AD, Kostov G, Athey B, Jones EG, Bunney WE, Myers RM, Speed TP, Akil H (2005). Evolving gene/transcript definitions significantly alter the interpretation of GeneChip data. Nucleic Acids Res.

[B54] The Microarray Lab at the University of Michigan. http://brainarray.mhri.med.umich.edu.

[B55] The BioConductor Project. http://bioconductor.org/.

