# Supplementary material for: Learning transcriptional regulatory networks from high throughput gene expression data using continuous three-way mutual information
Source: BMC Bioinformatics. 2008 Nov 3;9:467. doi: 10.1186/1471-2105-9-467 (PMC2613931; doi:10.1186/1471-2105-9-467)
Supplement: Additional file 1 — Supplementary figures, tables and notes. [file 1471-2105-9-467-S1.pdf]

Supplementary Figures

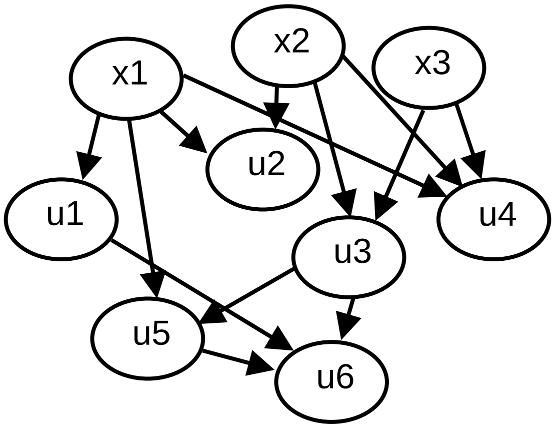

Supplementary Figure 1. Synthetic gene regulatory network. This synthetic model structure is designed to mimic a miniature gene regulatory network, with several major features. First the network contains a number of variables, 9 variables in total, 3 of which are independent and 6 dependent. Second, the variables are assembled into a hierarchy of regulatory relationships, with independent variable mimicking regulators and cofactors, and dependent variables mimicking target genes. Third, the complexity of the network is controlled in that dependent variables have 1-3 parents, mostly 2 or 3, and each regulator/cofactor controls a set of targets. Targets may share regulators and thus may have different levels of coregulation/coexpression, which can lead to confounding models. Fourth, a diverse set of continuous non-linear and logical relationships among variables were encoded by the algebraic formulas in Supplementary Table 1 to describe a realistic, yet complicated regulatory network.

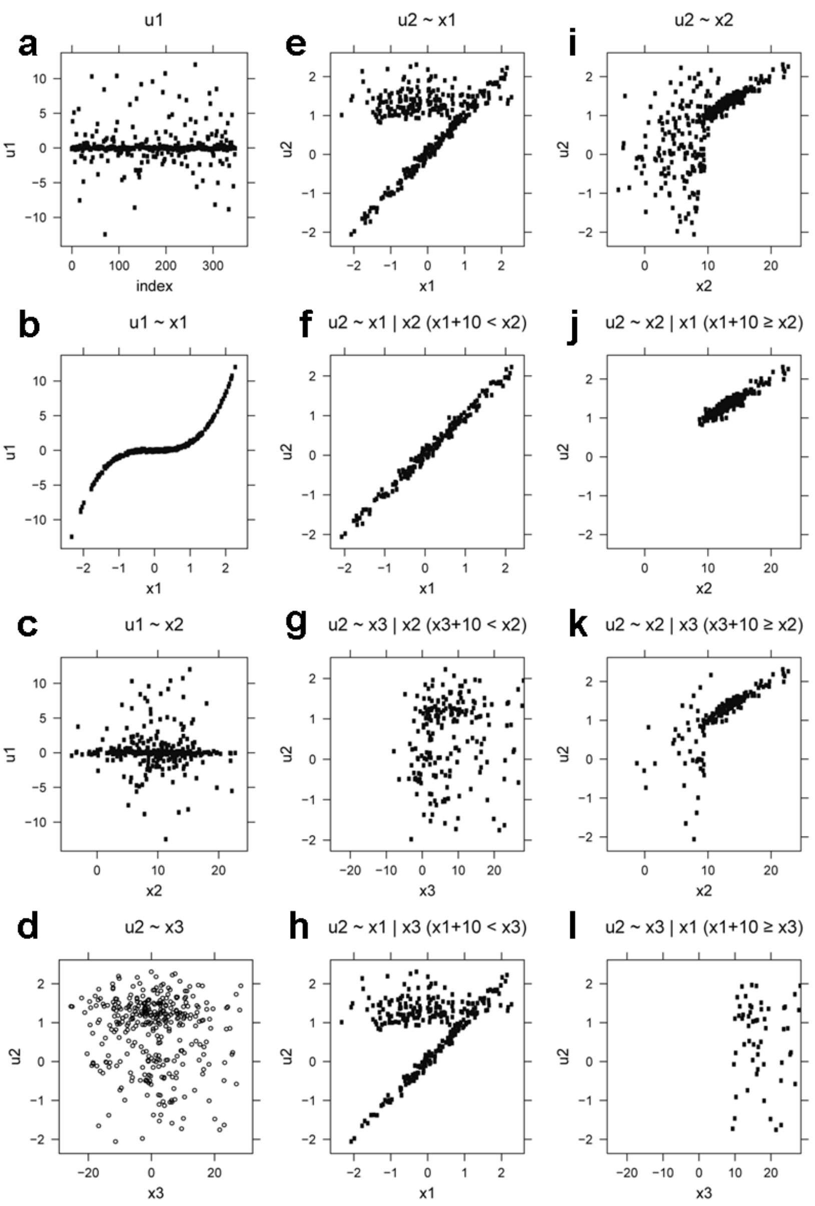

Supplementary Figure 2. Marginal and two-way joint distributions of the 350 data points sampled from the synthetic network (Supplemental Figure 1 and Supplemental Table 1) for nodes x1-3 and u1-3. (a-c) show the value of node u1 strongly depends on its causal node, x1, but not on control nodes like x2. (e, f, i, j) show the value of node u2 has limited marginal dependency on either on of its two causal nodes, x1 and x2, but has strong conditional dependency on either one of x1 and x2 given the other. We call this effect coordination between the causal nodes in determining the target node. (d, g, h, k, l) show no such coordination does not exists when we plug in a control node x3 in place of either one of the two causal nodes, x1 and x2.

Supplementary Tables

Variable	Algebraic formula	True parent set
x1	N(0,1)	
x2	N(10,5)	
x3	N(0,10)	
u1	$(x1)^3$ + N(0,0.1)	x1
u2	x1 + N(0,0.1), x1+10≥x2<br>x2/10 + N(0,0.1), $x1+10<x2$	x1, x2
u3	(x2-x3)/(x2+10) + N(0,0.05)	x2, x3
u4	x1+sin(x3) + N(0,0.1), x1+10≥x2<br>x2/10+sin(x3) + N(0,0.1), $x1+10<x2$	x1, x2, x3
u5	log(exp(x1)+exp(u3)) + N(0,0.1)	x1, u3
u6	(u1+u5)*u3/2+ N(0,0.05)	u1, u3, u5

Supplementary Table 1. Relationships encoded into the true models for the synthetic dataset. N(μ, σ) is a normal random distribution with mean of μ and standard deviation of σ.

(a)

One-way ANOVA	F	P		
	1712.35	0		
Tukey Test	MI3	BN	dMI3	MI2
MI3	NA	4.45E-11	4.45E-11	4.45E-11
BN	NA	NA	5.89E-11	4.45E-11
dMI3	NA	NA	NA	4.45E-11
MI2	NA	NA	NA	NA

(b)

One-way ANOVA	F	P		
	2818.09	0		
Tukey Test	MI3	BN	dMI3	MI2
MI3	NA	4.45E-11	4.45E-11	4.45E-11
BN	NA	NA	4.45E-11	4.45E-11
dMI3	NA	NA	NA	4.45E-11
MI2	NA	NA	NA	NA

Supplementary Table 2. One-way ANOVA followed by Tukey test on the performance for MI3 score and control scores in learning 2-parent models from synthetic data. (a) Testing statistics for average absolute sensitivity of the 4 methods when learning results were compared to true models; (b) Testing statistics for average relative sensitivity of the 4 methods when learning results were compared to best 2-parent models possible. Only testing results for sensitivities are shown, testing results for precisions are the same, since precision equals sensitivity multiplied by a constant of 1 (panel a) or 13/12 (panel b). F statistics and P-value are calculated for one-way ANOVA test, only upper triangle of the P-values table is shown for Tukey test (lower triangles would be symmetric to upper triangle). Tukey tests were conducted for all potential pair-wised comparisons, although of the most interest are those between MI3 versus control methods. Four scores: MI3, BN (log conditional probability), dMI3, MI2.

The experimental procedure is the same as Figure 2 in the main paper with only statistical test results for 350 data points are show here. Test results for other sample sizes are the same or very close. In the main paper, one representative experiment results at 350 data points (Figure 1), and average performance curves across different sample sizes, from 25 up to 1000, for all 4 scores are displayed (Figure 2).

Cutoff	Selected[†]	Verified	Verified Ratio
<0.1	8358	1156	0.138
0.1	4042	733	0.181
0.2	2226	513	0.230
0.3	1303	368	0.282
0.4	634	231	0.364
0.5	249	107	0.430
0.6	58	34	0.586
0.7	3	3	1.000

† MYC gene itself was pre-excluded from the target selection

Supplementary Table 3. Genes selected to be potential MYC targets (T) based on criterion I(MYC; T) > specific cutoff value: cutoff value vs total number of targets, number of targets verified against the MYC target database (http://www.myccancergene.org/), and the verified ratio.

Method	MI3		dMI3		BN		MI2	
Rank\R2	Symbol	Targets	Symbol	Targets	Symbol	Targets	Symbol	Targets
1	**ASH2L**	18	**PSIP1**	19	*PSMD14*	4	MRPL3	6
2	TRIP12	14	FNBP1	19	*SFRS1*	4	*PES1*	6
3	**ZNF143**	13	MRPL28	14	TXNDC9	3	*HSPC111*	6
4	ARPC1B	11	NIPSNAP1	7	PCID1	3	SSBP1	6
5	**CSK**	9	CD59	7	**GTF2A2**	3	**SSRP1**	6
6	**SIAH2**	7	RAB27A	6	*MSH2*	3	***MCM7***	5
7	FNBP1	6	ACOT8	5	NDUFAB1	3	TMEM53	5
8	**MIZF**	6	ARPC5	5	PSMA3	3	*JTV1*	5
9	**GCN5L2**	6	KIAA0922	5	KIF23	3	TPX2	4
10	PRPSAP1	5	**SIAH2**	5	*CHERP*	2	*MAD2L1*	4

Supplementary Table 4. Top 10 most frequently selected coregulators or MYC cofactors for 368 verified MYC targets with I(T; MYC) ≥ 0.3 by using MI3 or control methods: top 1 highest scoring cofactor is counted for each target. Cofactors in bold are involved in MYC dependent or general transcriptional regulation, those in italics are in the list of 368 verified MYC targets with I(T; MYC) ≥ 0.3. This table based on top 1 MYC cofactors are directly comparable to Table 2 in the main text based on top 5 MYC cofactors.

<table>
<tr><td>Relationship</td><td>OR</td><td>AND</td><td>XOR</td></tr>
<tr><td>Contigency Table</td>
<td><table><tr><td>p</td><td>R1</td><td>R2</td><td>T</td></tr><tr><td>1/4</td><td>0</td><td>0</td><td>0</td></tr><tr><td>1/4</td><td>1</td><td>0</td><td>1</td></tr><tr><td>1/4</td><td>0</td><td>1</td><td>1</td></tr><tr><td>1/4</td><td>1</td><td>1</td><td>1</td></tr></table></td>
<td><table><tr><td>p</td><td>R1</td><td>R2</td><td>T</td></tr><tr><td>1/4</td><td>0</td><td>0</td><td>0</td></tr><tr><td>1/4</td><td>1</td><td>0</td><td>0</td></tr><tr><td>1/4</td><td>0</td><td>1</td><td>0</td></tr><tr><td>1/4</td><td>1</td><td>1</td><td>1</td></tr></table></td>
<td><table><tr><td>p</td><td>R1</td><td>R2</td><td>T</td></tr><tr><td>1/4</td><td>0</td><td>0</td><td>0</td></tr><tr><td>1/4</td><td>1</td><td>0</td><td>1</td></tr><tr><td>1/4</td><td>0</td><td>1</td><td>1</td></tr><tr><td>1/4</td><td>1</td><td>1</td><td>0</td></tr></table></td></tr>
<tr><td>H(T)</td><td>$2-0.75*\log_2 3$</td><td>$2-0.75*\log_2 3$</td><td>1</td></tr>
<tr><td>H(R1)=H(R2)</td><td>1</td><td>1</td><td>1</td></tr>
<tr><td>H(T,R1)=H(T,R2)</td><td>1.5</td><td>1.5</td><td>2</td></tr>
<tr><td>H(R1,R2)</td><td>2</td><td>2</td><td>2</td></tr>
<tr><td>H(T,R1,R2)</td><td>2</td><td>2</td><td>2</td></tr>
<tr><td>I(T;R1)=I(T;R2)=
H(T)+ H(R1)- H(T,R1)</td><td>$1.5-0.75*\log_2 3$</td><td>$1.5-0.75*\log_2 3$</td><td>0</td></tr>
<tr><td>I(T;R1,R2)= H(T)+
H(R1,R2) -H(T,R1,R2)</td><td>$2-0.75*\log_2 3$</td><td>$2-0.75*\log_2 3$</td><td>1</td></tr>
<tr><td>I(T;R1,R2)-
I(T;R1)-I(T;R2)</td><td>$0.75*\log_2 3-1$
=0.189</td><td>$0.75*\log_2 3-1$
=0.189</td><td>1</td></tr>
</table>

Supplementary Table 5. The non-additive property of high order interactions, i.e. I(T;R1,R2)-I(T;R1)-I(T;R2) = I(T;R1;R2) >0, shown by common types of regulatory relationships involving two independent parents (R1 and R2) and a target (T). Entropies (H's) and mutual information (I's) are calculated according to definitions in Supplementary Note 1. These are ideal cases. In reality, we don't always get positive high order interactions due to the data quality and absence of real regulators in the data. Hence we don't impose any threshold on high order interaction alone.

Supplementary Notes

Supplementary Note 1: Mutual information definition, extension and calculation

Here we describe entropy and mutual information definition for discrete variables. The corresponding definition for continuous variables remained the same [1], except that the summation becomes integration in the following formulas.

In information theory, for a discrete variable, X, Shannon entropy H(X) is defined to be [2]:

$$H(X) = -\sum_{i=1}^{Mx} P(x_i)\log_2 P(x_i) \tag{1}$$

Where $X=x_i$ (i=1,2, , M_x), corresponding to M_x different states of variable X, notice that M_x may be different from total number of data points. Shannon entropy is a measurement for the randomness of variable distribution, i.e. how unpredictable the value or state of a variable is. The higher the Shannon entropy is, the harder to predict the value or state of this variable. Similarly, the entropy of joint distribution of two discrete variables X and Y is defined to be [2]:

$$H(X,Y) = -\sum_{i=1}^{Mx}\sum_{j=1}^{My} P(x_i, y_j)\log_2 P(x_i, y_j) \tag{2}$$

Where $Y=y_j$ (j=1,2, , M_y), corresponding to M_y different states of variable Y.

Mutual information between two variable X and Y, I(X;Y), is defined based on Shannon entropy, it equals the difference between the sum of entropy of X and Y individually vs the entropy of them jointly [2, 3]:

$$I(X;Y) = H(X) + H(Y) - H(X,Y) \tag{3}$$

Mutual information measures the difference in predictability when considering two variables together versus considering them independently. Said another way, mutual information is a measurement of dependency between variables. High dependency or mutual information usually occurs when there is causal relationship between variables, or common causal factors exit. Therefore, mutual information can be used to identify best predictors, or even causal factors and target/dependent factors of variables.

One specific problem addressed in this work is the mutual information among multiple variables. We extended entropy and mutual information definitions in formula (1-3)

correspondingly. For 3 variables X, Y, Z, we can define three types of three-way mutual information: total correlation C(X;Y;Z) [4], generalized two-way I(X;Y,Z), and three-way interaction information I(X;Y;Z) [5, 6]:

$$C(X;Y;Z) = H(X) + H(Y) + H(Z) - H(X,Y,Z) \tag{4}$$

$$I(X;Y,Z) = H(X) + H(Y,Z) - H(X,Y,Z) \tag{5}$$

$$I(X;Y;Z) = H(X,Y) + H(Y,Z) + H(X,Z) - H(X) - H(Y) - H(Z) - H(X,Y,Z) \tag{6}$$

These are all generalized mutual information of order 3, different in lower order terms:

$$I(X;Y,Z) = C(X;Y;Z) - I(Y;Z) \tag{7}$$

$$I(X;Y;Z) = I(X;Y,Z) - I(X;Y) - I(X;Z) \tag{8}$$

Supplementary Table 5 show common examples, where the relationships are high order and can only be fully captured by high order mutual information.

Conditional entropy and mutual information can also be defined based on conditional probability. A rearranged version of conditional mutual information can derived by starting with the definition of conditional probability given Z:

$$I(X;Y \mid Z) = \frac{1}{N}\sum_{k=1}^{N}\log_2 \frac{P(x_k, y_k \mid z_k)}{P(x_k \mid z_k)P(y_k \mid z_k)} \tag{9}$$

Next, apply Bayes' rule and rearrange to yield:

$$I(X;Y \mid Z) = \frac{1}{N}\sum_{k=1}^{N}\log_2\left[\frac{P(x_k, y_k, z_k)}{P(x_k)P(y_k, z_k)}\frac{P(y_k, z_k)}{P(y_k)P(z_k)}\right] \tag{10}$$

Re-write into mutual information:

$$I(X;Y \mid Z) = I(X;Y,Z) - I(X;Z) \tag{11}$$

Apparently, this conditional mutual information is of order 3 and is closely related to all other types of three-way mutual information. So far, we have been focusing on three-way mutual information and entropy. Similarly, the conception of entropy and mutual

information can be directly extended to arbitrary higher order to capture even complicated relationships among multiple variables or multiple sets of variables.

Supplementary Note 2: Comparison between MI and log-based local conditional probability

Plug entropy definitions formula (1) and (2) into formula (3), we get the expanded formula for mutual information based on probability:

$$I(X;Y)=\sum_{i=1}^{Mx}\sum_{j=1}^{My}P(x_i,y_j)\log_2\frac{P(x_i,y_j)}{P(x_i)P(y_j)}=\frac{1}{N}\sum_{k=1}^{N}\log_2\frac{P(x_k,y_k)}{P(x_k)P(y_k)} \tag{12}$$

Where X=x_k (j=1,2, , N) Y=y_k (j=1,2, , N), corresponding to N data points of variable X or Y.

The counterpart to mutual information in Bayesian network (BN) is log-based local conditional probability, or log likelihood (LL) can be expanded as:

$$LL(X\mid Y)=\log\prod_{k=1}^{N}P(x_k\mid y_k)=\sum_{k=1}^{N}\log\frac{P(x_k,y_k)}{P(y_k)} \tag{13}$$

It can be seen that mutual information is close to log likelihood. However mutual information is more standardized, with a weighted-averaging term 1/N and normalizing term P(x_k), which minimize the effects of sample size and specific distribution of individual variables. Without these two terms, log likelihood decreases with larger samples size N and fluctuate greatly with the individual distributions of X and Y, and it becomes difficult to compare models with different data sizes and variables/nodes. Therefore, mutual information is a score better tailored for local model/network learning than log likelihood.

Supplementary Note 3: presentation of probabilistic causation using directed graphs

Directed graphs, including directed acyclic graphs (DAG) or Bayesian Networks, have been well established tools for probabilistic causation modeling [7]. In these graphs, directed arrows between nodes represent causal relationships. For instance, the synthetic network (Supplementary Figure 1) shows node x1 cause u1. This relationship can be interpreted as the value of variable x1 (event A: x1 = a specific value) directly determines/alters on the probability distribution of the variable u1 (probability of event B: u1 = a specific value). Indeed, x1 strongly affects u1 distribution (Supplementary Figure 2b), whereas another node x2 has almost no effect on u1 (Supplementary Figure 2c). From a predictive point of view, knowing the value of x1 greatly narrows down (helps

predict) the potential values of u1 (Supplementary Figure 2b vs 2a) while knowing the value of x2 helps very little if any (Supplementary Figure 2b vs 2a). This is a simple two-way causal relationship, where there is one causal node and one target node. The synthetic network and real gene regulatory network features high-order causal relationships, where two or more other nodes cause one target node. In the synthetic network (Supplementary Figure 1), x1 + x2 -> u2 is an example of three-way causal relationship. Either one of the two causal nodes, x1 or x2, does affect (and predict) the distribution of u2 marginally, yet to a limited extend (Supplementary Figure 2e and 2i). But both nodes jointly tell a lot more on u2. In other words, knowing x2, x1 can predict u2 extremely well, and vice versa (Supplementary Figure 2f vs 2e, 2j vs 2i). We call this effect coordination or synergy between causal factors in determining the target (more description in Methods). As a control case, knowing x3, a fake causal factor of u2 does not help at all on the prediction of u2 either using x1 or x2 (Supplementary Figure 2h vs 2e, 2k vs 2i). Similarly, knowing x1 or x2 does not help predicting u2 using x3 either (Supplementary Figure 2g vs 2d, 2l vs 2d).

Clearly, these high-order causal relationships are more complicated than two-way relationships hence cannot be fully measured by two-way or correlative metrics (Supplementary Figure 2e vs 2d, 2i vs 2h, more examples are given in Supplementary Table 5). In this paper, we propose to capture high-order causal relationships using a high-order mutual information based metric, MI3 (Methods and Supplementary Table 5). MI3 effectively differentiate causal vs confounding relationships, where two-way or correlative metrics fail frequently (Figure 4 and 5).

Supplementary Note 4: Exhaustive search for the best R1-R2 pairs given T, but not the best R2-T pairs given R1

In MI3, model learning was focused locally, i.e. we scored and compared all possible local regulatory models for specific target T. This target centered model learning applied to both synthetic data and experimental data, even though biologically we are interested in constructing models centered at particular R1=MYC in the latter case. It would be less appropriate to compare models across different T's because they are not mutually exclusive. Similarly, in Bayesian network, logP(T|R1,R2) is only comparable for fixed T, where all other terms including P(R1)P(R2) in the full product form of joint probability [8, 9] cancelled out. Therefore, we only searched for best R1-R2 pairs given T, but not best R2-T pairs given R1 when learning probabilistic models based on MI3 score or log conditional probability or any other established score. This local approach makes it affordable for MI3 to conduct exhaustive search, which leads to globally optimized models. Heuristic search can be taken when computing time is limited.

Supplementary Note 5: Major differences in learning regulatory models from microarray

data versus synthetic data

When MI3 is applied to an experimental gene expression dataset, two key differences between experimental data and synthetic data need to be considered. First, in our gene expression data there are 8359 genes, which is significantly larger system than the 9-variable synthetic network. For an exhaustive search for the best two-parent set for each gene, this problem size would require searching ~10^{11} (8359^3) combinations—a scale that is currently out of reach computationally. In this work, we focus on the construction transcription regulatory networks centered to MYC. Therefore, we can fix one regulator, R1, to MYC, and only search across cofactors (R2s) and targets (T). This reduced problem requires the search of ~10^7 (8359^2) combinations for our gene expression data. This scale of problem is computationally tractable. For both scenarios, we constrain MYC targets (T) with I(T; MYC) ≥0.3, i.e. targets that have enough marginal dependency on MYC to ensure that MYC does likely regulate the target based on the the microarray dataset. Second, there are frequently multiple equally interesting and closely scoring regulatory models learned from experimental data for each target. For example, several regulators are equally important, or multiple genes in a pathway/complex represent the same regulatory action equally well. Correspondingly, we kept the top 5 highest scoring 2-parent models for each target gene, rather than the top 1 as in the synthetic data. Keeping top 1 model only led to almost the same list of top 10 MYC cofactors (Supplementary Table 3-4), except that the number of targets mapped to individual cofactors was too small for quantitative evaluation.

Supplementary References

1. Steuer R, Kurths J, Daub CO, Weise J, Selbig J: **The mutual information: detecting and evaluating dependencies between variables**. *Bioinformatics* 2002, **18 Suppl 2**:S231-40.
2. Shannon CE: **A Mathematical Theory of Communication**. *Bell System Technical Journal* 1948, **27**:379-423.
3. Kolmogor.An: **Logical Basis for Information Theory and Probability Theory**. *Ieee Transactions on Information Theory* 1968, **IT14**:662-&.
4. Watanabe S: **Information Theoretical Analysis of Multivariate Correlation**. *Ibm Journal of Research and Development* 1960, **4**:66-82.
5. Jakulin A, Bratko I: **Quantifying and Visualizing Attribute Interactions: An Approach Based on Entropy**. *arXiv:cs.AI/0308002* 2004.
6. Mcgill WJ: **Multivariate Information Transmission**. *Psychometrika* 1954, **19**:97-116.
7. Pearl J: **Causality : models, reasoning, and inference**. Cambridge, U.K. ; New York: Cambridge University Press; 2000.
8. Friedman N, Linial M, Nachman I, Pe'er D: **Using Bayesian networks to analyze expression data**. *J Comput Biol* 2000, **7**:601-20.
9. Hartemink AJ, Gifford DK, Jaakkola TS, Young RA: **Using graphical models and genomic expression data to statistically validate models of genetic regulatory networks**. *Pac Symp*

Biocomput 2001:422-33.
